# Barriers and facilitators of adherence to antidepressants among outpatients with major depressive disorder: A qualitative study

**DOI:** 10.1371/journal.pone.0179290

**Published:** 2017-06-14

**Authors:** Siew Ching Ho, Sabrina Anne Jacob, Balamurugan Tangiisuran

**Affiliations:** 1 School of Pharmaceutical Sciences, Universiti Sains Malaysia, Penang, Malaysia; 2 School of Pharmacy, Monash University Malaysia, Selangor, Malaysia; 3 WHO Collaborating Centre for Drug Information, National Poison Centre, Universiti Sains Malaysia, Penang, Malaysia; TNO, NETHERLANDS

## Abstract

**Background:**

One of the major challenges in treating major depressive disorder (MDD) is patients’ non-adherence to medication. This study aimed to explore the barriers and facilitators of patients’ adherence to antidepressants among outpatients with MDD.

**Methods:**

Semi-structured and individual in-depth interviews were conducted among patients with MDD who were taking antidepressants, in the psychiatric clinic of a government-run hospital in Malaysia. Participants were purposively sampled from different genders and ethnicities. Interviews were conducted using a validated topic guide, and responses were audio-recorded, transcribed verbatim, checked, and analyzed using the grounded theory approach.

**Results:**

A total of 30 patients were interviewed. Forty different themes and sub-themes were identified which were conceptually divided into two distinct categories related to barriers and facilitators to adherence. The barriers were: patient-specific, medication-specific, healthcare provision and system, social-cultural, and logistics. The facilitators were: having insight, perceived health benefits, regular activities, patient-provider relationship, reminders, and social support networks.

**Conclusions:**

Patient-specific barriers and medication side effects were the major challenges for adhering to treatment. Perceived health benefits and having insight on the need for treatment were the most frequently cited facilitators. Targeted interventions should be developed to address the key barriers, and promote measures to facilitate adherence in this group of patients.

## Background

Major depressive disorder (MDD) is a major burden for the health care system worldwide in terms of health-related quality of life, medical morbidity and mortality, and increased utilization of health care services [1–3]. MDD was rated as the fourth leading disabling condition [2], and according to projections for the year 2030, it will rise to be among the top three leading disabling conditions together with ischemic heart disease and HIV/AIDS [4]. In Malaysia, MDD is the most common mental illness reported [5]. The National Health Morbidity Survey IV in 2011 showed that the prevalence of lifetime depression was 2.4%, and the current rate of depression among adults was 1.8%, which translates to approximately 0.3 million adults in Malaysia [6].

Antidepressants (ADs) are the cornerstone of MDD treatment and have been proven to effectively reduce depressive symptoms [7, 8]. However, non-adherence to ADs remains a common problem and has been widely recognized as one of the reasons for treatment failure in MDD; with the evidence reporting that rates of non-adherence vary from 10 to 60% [9–12]. A recent systematic review showed that non-adherence to ADs is associated with poorer clinical and economic outcomes such as increased risk of relapse and recurrence, increased emergency department visits and hospitalization rates, as well as increased economic burden on the healthcare system [13]. A study conducted in the United States (US) found that of all the medication-related admissions, 33–69% was due to poor medication adherence, with a resultant cost of approximately USD 100 billion per annum [14]. Hence, a better understanding on the barriers to adherence in patients with MDD is crucial in order to identify effective interventions to facilitate adherence, and to improve the clinical and economic outcomes in these patients [15, 16].

Several studies have reported on the factors that lead to non-adherence such as patients’ social and demographic characteristics, education level, medication side effects, poor patient-clinician interaction, negative attitudes, and culture–influenced beliefs [17–23]. Of all these factors, patients’ attitudes and personal beliefs regarding MDD and its treatment are thought to differ significantly between different cultures. Using the Antidepressant Compliance Questionnaire (ADCQ), Kessing et al [24] assessed the attitudes and beliefs of patients with depression and bipolar disorder towards their disease and its treatment. The study found that up to 80% of patients had erroneous beliefs regarding the effects of ADs. Horne et al. [25] conducted a cross-sectional study using the Beliefs about Medication Questionnaire, involving 83 Asians and 417 Europeans. Results showed that Asians had more negative attitudes and beliefs about medications compared to the Western population, and believed that prolonged use of medications could lead to harm and addiction. Malaysia is a multiracial country made up of 60% Malays, 20% Chinese, and 10% Indians. Hence, it is important to study the reasons why patients do or do not adhere to ADs by taking into account patients’ attitudes and beliefs, given the impact of different cultures and religions on patients’ drug-taking behaviour.

Most of the qualitative studies undertaken so far have mainly explored patients’ experience in relation to other health problems such as hypertension [26] and antiretroviral drug therapy [27], but there are limited qualitative studies on mental health disorders such as depression. Other studies conducted thus far have been quantitative in nature [28–32], however using a structured set of close-ended surveys have not allowed patients to fully express themselves and to give responses outside the options given on the questionnaires. To date, there have also been relatively few studies conducted in Malaysia that have focused on patients’ experience with using ADs. Therefore, this study aims to fill that gap by exploring, in-depth, the barriers and facilitators of adherence to ADs in patients with MDD using a qualitative method. Understanding the attitudes and reasons for their behaviour can provide valuable insights into identifying problems with adherence. Results obtained from the study will be of significance to develop strategies or interventions that can help address these problems, and to design better adherence programs that will cater not only for the Malaysian setting and culture, but also worldwide.

## Methods

### Study design

A qualitative grounded theory methodology was adopted as it was deemed appropriate to explore patients’ experiences and views on the use of ADs [33, 34]. This study was approved by the Medical Research and Ethics Committee [reference number NMRR-14-1703-22855 (IIR)] of the Ministry of Health, Malaysia.

### Participant selection

The number of participants recruited was based on data saturation, that is, data were collected until no new themes were identified and no issues emerged from the interviews [35, 36]. Generally, 15–20 participants are considered sufficient to achieve data saturation [37]. However, larger sample sizes can be used to confirm the themes obtained [38]. In this study, the researcher intentionally over-sampled to obtain equal numbers of participants based on gender and ethnicity. Equal numbers of males and females from each ethnic group, who met the inclusion and exclusion criteria, were recruited into the study. Despite this measure taken, the study was not designed to focus on the comparison between demographic characteristics, thus, the study outcome will not be affected. Purposive sampling was used to recruit patients according to the following inclusion criteria: All patients aged 18 years and above diagnosed with MDD by the treating physicians from the psychiatric department, based on the Diagnostic and Statistical Manual of Mental Disorders IV (DSM-IV) [39] or according to the International Classification of Diseases-10 (Mental and Behavioural Disorders, ICD-10); regardless of severity, and who were currently taking any class of oral ADs for at least six months, regardless of their adherence pattern or change of treatment regimen. The following patients were excluded: patients with a co-morbid psychiatric diagnosis such as schizophrenia or bipolar disorder during the study period, patients with dementia, impaired cognitive abilities, mental retardation, Alzheimer’s or Parkinson’s, and patients who were unable to understand or speak either English, Bahasa Malaysia (the national language) or the Chinese dialect of Mandarin.

Patients who agreed to participate and signed the informed consent forms were assured that results would only be presented in de-identified terms. Demographic data and relevant clinical information of each patient were captured and recorded using a validated standardised data collection form.

### Study setting

Patients were recruited from the out-patient psychiatric clinic of a government-run hospital in the Klang Valley in Malaysia. The clinic has an average of 1600 patients a month, and MDD is the most commonly treated psychiatric disorder in the clinic.

### Data collection

A semi-structured interview was conducted using a list of pre-determined open-ended questions that was developed based on previously published qualitative research, as well as the objectives of the study (S1 File). Face and content validity was done by experts in mental health and questionnaire development. Most of the questions were open-ended to encourage participants to provide in-depth information. Individual interviews with patients were carried out in a private room to ensure confidentiality [38]. The average time for each interview session was 45 minutes and the interviews were conducted in English, Bahasa Malaysia or Mandarin, according to the patient’s preference. Probing questions were asked to provoke the respondents and to further explore the answers in-depth. The interviews were audio-recorded and transcribed verbatim. All translations were done by a certified translator using Brislin’s backward forward translation [40]. A pilot study was conducted involving three patients to improve consistency, and to ensure the interview questions were clear and relevant. The pilot study showed that patients were able to understand and answer the interview questions clearly. Therefore, the interview topic guide was maintained and used for subsequent interviews. Data obtained from the pilot study were not included in the final analysis.

### Data analysis

Coding was performed manually and line-by-line to identify initial phenomena, and to produce a list of themes that were of importance to the study. The themes obtained from each interview transcript were carefully listed, checked, compared constantly, and refined in order to choose and identify the most telling codes to represent the interviewees’ voice; which is known as focused codes. Coding and thematic analysis was conducted by the researcher independently. However, the themes obtained were reviewed and verified by the other researchers to increase the validity of the findings. Any coding discrepancies were resolved by consensus. Focused codes that shared similar characteristics were pooled together and merged into more abstract categories which were interlinked, and built the basis of a theory to answer the research questions [41]. Quotations by respondents were edited on a limited basis to remove content that did not convey meaning (repeated words, stutters) and to correct for grammar. An ellipsis mark was used to note removal of such extraneous content. Square brackets were used in quotations to supply words omitted by the speaker.

## Results

A total of 49 patients with MDD were approached to participate in this study. Of this, only 30 patients were included and interviewed. Reasons for non-participation included no interest (n = 7), time factor (n = 6), and data collection having reached saturation (n = 6). The baseline characteristics of the included patients are shown in Table 1. The mean age was 45.2± 9.6 years, ranging from 27 to 59 years; with an equal number of patients from both genders. Malay, Chinese, and Indian patients were distributed evenly. Patients were predominantly married, and the majority had secondary school as their highest level of education. The duration of MDD and use of ADs was highly variable, with a mean duration of 8.8 years, ranging from seven months to 33 years.

**Table 1:** Patients demographic and clinical background.

Demographic characteristics	Number of participants (%)
Age (years)	45.2± 9.6*
**Sex**	
Male	15 (50)
Female	15 (50)
**Ethnicity**	
Malay	10 (33.3)
Chinese	10 (33.3)
Indian	10 (33.3)
**Religion**	
Islam	10 (33.3)
Buddhist	8 (26.7)
Hindu	8(26.7)
Christian	4 (13.3)
**Current marital status**	
Single	2 (6.7)
Married	22 (73.3)
Divorced	6 (20)
**Current monthly household income (RM)**	
<1500	6(20)
1501–2500	10 (33.3)
2501–3500	5 (16.7)
3501–4500	5(16.7)
>4500	4 (13.3)
**Highest level of education**	
Primary school	2 (6.7)
Secondary school	21 (70)
Diploma	3 (10)
University or higher	4 (13.3)
**Current employment**	
Unemployed	2 (6.7)
Housewife	6 (20)
Self-employed	1 (3.3)
Retired	2 (6.7)
Others	19 (63.3)
**Duration of MDD (years)**	8.8±8.6*
**Duration of AD used (years)**	8.8±8.6*
**Use of AD**	
SSRIs	23 (76.7)
SNRIs	2 (6.7)
Miscellaneous	5 (16.7)
**Other medications**	
Benzodiazepine	7 (23.3)
Others (Antihypertensive etc)	14 (46.7)
Not taking any other medication	9 (30)
**Co-morbidities**	
Hypertension	7 (23.3)
Diabetes	2 (6.7)
Asthma	3 (10)
Cardiovascular disease	3 (10)
Others (Liver disease, BPH and etc)	4 (13.3)
No co-morbidities	15 (50)

SD: Standard deviation, RM: Ringgit Malaysia (1 USD = RM4.44), MDD: Major depressive disorder, ADs: Antidepressants, SSRIs: Selective Serotonin Reuptake Inhibitors, SNRIs: Selective Norepinephrine Reuptake Inhibitors, BPH: Benign prostatic hyperplasia

*Data was presented using mean and SD.

Analysis of all interview transcripts identified 40 different themes and sub-themes, which were conceptually divided into five categories of barriers (Table 2), and six categories of facilitators (Table 3).

**Table 2:** Summary of categories, sub-categories and themes for barriers of adherence to antidepressants in patients with MDD.

Categories	Sub-categories	Themes
1. Patient-specific barriers	1.1. Erroneous beliefs	• Misconceptions about MDD and/or ADs
		• Fear of drug dependence
	1.2. Forgetfulness	• Having a busy schedule
		• Being away from home
		• Simply forgetting to take their ADs
	1.3. Negative attitudes	• A dislike for the pills
	1.4. Co-morbidity	• Alcohol dependence
	1.5. Lack of knowledge	• About the use of ADs
		• About the effect of ADs
2. Medication-specific barriers		• Side effects
		• Pill burden
		• Treatment duration
		• Costs of medications
3. Healthcare provision and system		• Multiple prescribers
		• Problems communicating with healthcare providers
		• Long waiting time at the clinic
		• Frequent medication refills
		• Frequent clinic visits
		• No supply of medications
4. Social-cultural barriers		• Lack of support from family/spouse/friends
		• Barriers related to religion and cultural beliefs
		• Stigma
5. Logistic barriers		• Poor access to healthcare locations

MDD: major depressive disorder; ADs: antidepressants

**Table 3:** Summary of categories, themes, and sub-themes for facilitators of adherence to antidepressants in patients with MDD.

Categories	Themes	Sub-themes
1. Illness-related factors	1.1. Insight	• Wish for complete recovery
		• Fear of relapse
		• Experience of recurrence
		• Awareness about the need to take ADs
2. Perceived health benefits	2.1. Positive beliefs about ADs	
	2.2. Effectiveness of ADs	
3. Regular activities	3.1. Taking with a meal	
	3.2. Daily routine	
4. Patient-provider relationship	4.1. Trusting healthcare providers	
	4.2. Desire to please the healthcare providers	
	4.3. Fear of healthcare providers	
5. Reminders	5.1. Using pillboxes	
	5.2. Reminder from family members	
	5.3. Keeping medications in visible places	
6. Social support networks	6.1. Support from family members/spouse/ children/friends/co-worker	
	6.2. Responsibility toward family members	

ADs: antidepressants

### Barriers of adherence to antidepressants

Barriers are difficulties or problems that patients face when taking their ADs as prescribed by the physician. Five major categories were identified: patient-specific, medication-specific, healthcare provision and system, social-cultural, and logistics.

#### Category I: Patient-specific barriers

Patients-specific barriers emerged as the most important category, reported by 83% of participants. They were further sub-categorized into erroneous beliefs, forgetfulness, negative attitudes, lack of knowledge, and co-morbidity. For the subcategory of erroneous beliefs, there were many misconceptions noted either about the disease itself or regarding the medications. The majority of Malay and Chinese patients feel there is no need to take medications in the absence of symptoms of depression. Patients also believe that they can simply discontinue their ADs when they feel better.

“I do not take my medication when I am well. I will only continue taking it (AD) when I am sick or depressed.” (Female, Malay #1)

Patients believe that ADs are toxic and may cause kidney damage if taken long term, as cited by 50% of participants. There is also a concern on the addictive and dependent potential of ADs, which further hampered their adherence.

“People always say that taking antidepressants will cause other things (illness) or kidney damage. It will usually cause side effects on our kidney, especially if we take the medication for long term.” (Female, Malay #4)

Patients also admitted to sometimes forgetting to take their medication due to being busy, away from home, or due to forgetfulness.

“Two years ago, when I was taking care of my mother, she was sick and went on dialysis. I still needed to take care of my son besides taking care of my mother… I was really busy. At that time I did miss my dose. It’s not [that] I did not want to take it, but it’s because I was too busy.” (Male, Malay #2)

Negative attitudes were identified as another barrier, where attitudes are defined as patients’ emotional responses with regard to MDD and its treatment. Having a dislike of pills was identified by patients as one of the barriers to adherence. Besides that, the presence of co-morbidities such as alcohol dependence may influence patients’ adherence to medication as patients admitted that they would usually discontinue their medication so that they can drink alcohol.

“I was an alcoholic. [After] I started taking this medicine, after some time, I would go back to alcohol and stop it (AD). I would just throw all my medicine away.”(Male, Indian #3)

A lack of knowledge about MDD and its treatment was also associated with non-adherence, as reported by 13% of patients.

“Sometimes I don’t even know why I am taking it (AD). [If I can] sleep, then I am okay. I [am able to] sleep even without taking this medication. So, why should I take it?” (Female, Indian #2)

#### Category II: Medication-specific barriers

Medication-specific barriers were highlighted by 63% of patients, and consists of four main themes namely side effects, pill burden, treatment duration, and cost of treatment. The majority of patients reported that they had experienced significant side effects with ADs, which caused them to stop taking their medication. Indeed patients would adjust their medication or stop completely due to the side effects they experienced, without prior discussion with their physicians. The most common side effect reported was drowsiness (23%), followed by fatigue, and sexual dysfunction.

“The drowsiness was really bad. I'll only take it (AD) when I cannot sleep or when I feel stressed. I have reduced the dose…by myself. Sometimes I [only] take [the tablet] once in three days because of the drowsiness.”(Male, Malay #4)

Patients with co-morbidities mentioned pill burden as a barrier as they felt tired of taking so many types of medications, causing them to choose to discontinue their ADs instead.

“…I have heart problems, so…every day I have so many medications to take. If I could choose, I would skip this (AD).”(Female, Chinese #1)

Many patients continued to question the treatment duration of ADs, with some expressing concerns about committing to long-term treatment for their MDD.

“When can I actually…stop (taking ADs)? This is the question that I always want…the answer [to] I do not want to take it for long term.” (Male, Chinese #2)

Cost emerged as the least mentioned barrier in the study, with only two patients reporting worries related to the costs of medications.

“I stopped going for my doctor’s appointment because the private doctor is very expensive…I can’t afford to continue my treatment there.” (Male, Chinese #3)

#### Category III: Healthcare provision and system

There were six main themes identified in this category: multiple prescribers, problems communicating with healthcare providers, long waiting time at the clinic, frequent medication refills, frequent clinic visits, and no supply of medications. Multiple prescribers reduced patients’ confidence in the treating physician, subsequently influencing their medication-taking behaviour.

“The doctor kept changing. If every time we see the same one, we would have more confidence in that doctor and will continue the treatment.” (Male, Indian #5)

Patients reported that they tend to not inform their physicians about altering their medication, or discuss their illness and its treatment with their physicians, due to communication problems.

“I will not take my pill exactly as prescribed by the doctor. I will try to adjust it accordingly. I felt my depression was much better after I adjusted my pill by myself. I have told [the] doctors about it before but they just don’t care, and asked me to take it (AD) as directed. So [now], I also don’t bother telling them.” (Male, Chinese #5)

Patients also sometimes intentionally skipped their clinic appointments, lamenting the long waiting times at the clinic, and frequent clinic visits for dosage adjustment. This then resulted in patients not taking their medications due to insufficient amounts at home.

“A lot of patients at the clinic…we need to wait. Normally…about half a day. So, sometimes I will miss my appointment and not take my medicine.” (Female, Chinese #2)

Other barriers include a lack of supply of medications, and the need for frequent visits for medication refills due to the fact that most pharmacies in government hospitals in Malaysia adopt a one-month supply policy.

“I think the only difficulty for me in taking this medicine is the supply of it. If possible, give us enough medication [to last] until the next appointment. It would save us the hassle of having to come to the hospital every month [for a refill].” (Male, Malay #3)

#### Category IV: Social-cultural barriers

Lack of support, stigma, and barriers related to religion and cultural beliefs were themes that emerged as social-cultural barriers to adherence. Unhelpful family members, especially spouses, were found to discourage patients from continuing their medication. Patients also mentioned that fear of stigma influenced their adherence.

“My family members told me not to take this medicine. They said it’s not good to take so many medications especially for long term…so I don’t take it.” (Male, Chinese #1)

“I do not disclose (my illness) to my friends and family. My friends think I am alright because I don’t bring my medication when I go to meet them.” (Male, Indian #1)

With regard to the impact of patients’ religious and cultural beliefs, patients noted that if being prayerful would cure them of depression, then that would be an impetus to stop their ADs. As mentioned by a Buddhist patient:

“I have thought about stopping (my) antidepressants…if praying to Buddha or reading Buddhist-related books can help me…can solve my sleeping problem.” (Male, Chinese #1)

Similarly, a Hindu female patient said:

“If prayers can make me okay…no more depression… then I do not want to take medication.” (Female, Indian #3)

#### Category V: Logistic barriers

Logistic barriers were defined as issues related to inconveniences that hindered patients from adhering to their ADs, and include poor access to healthcare locations.

“I want to collect my medication (refill) at the clinic near my house. That would be more convenient for me because I have no transport to come to the hospital, so sometimes I do not take my medications.” (Female, Malay #3)

### Facilitators of adherence to antidepressants

Facilitators are defined as situations or factors that assist patients in adhering to their ADs, as prescribed by the physician. Six major categories were identified: illness-related factors, perceived health benefits, regular activities, patient-provider relationship, reminders, and social support networks.

#### Category I: Illness-related factors

Insight emerged as one of the main themes, cited by 93% of patients. Having insight was further sub-categorized into the wish for complete recovery, fear of relapse, experience of recurrence, and awareness of the need to take ADs. Patients reported that the wish to gain complete recovery, and fear of relapse encouraged them to adhere to their medications. Patients were afraid that their MDD would get worse if they did not continue with their medication. Indeed, one third of patients reported that they had experienced a relapse or an increase in symptom severity, as a result of discontinuing their ADs without prior discussion with their physicians.

“I just want to take my medication so that I will recover from my depression.” (Female, Indian #5)

“I am afraid I will get sick again if I don’t take it. I do not dare to stop (the medicine). I fear that my depression will come back again. I do not want to be depressed again.” (Female, Malay #5)

Most of the patients appeared to have an awareness about the need for treatment, and acknowledged that they were sick and in need of treatment.

“We must be very clear that we have a health problem now and we need medication to recover from the illness. If we are sick and have to take a lot of medication, we have to take it. If we want to get well, we have to take medication.” (Male, Malay #3)

#### Category II: Perceived health benefits

Perceived health benefits referred to patients’ assessment of the efficacy of ADs, and the value of taking ADs to reduce their depressive symptoms. Positive beliefs about ADs and its effectiveness were the two themes that emerged in this category, with patients expressing high trust in the ability of ADs to cure their MDD.

“I can see the effect of the medicine that I am taking. It did help me to get well. I know the medicine is helpful, it’s effective. So I will automatically keep taking it.” (Female, Chinese #4)

#### Category III: Regular activities

Regular activities are activities that patients engaged in regularly to help them incorporate medication-taking into their daily activities. Taking medication with a meal was something that facilitated patients’ adherence to ADs.

“I will take my antidepressant after my dinner. I will do it right away without waiting…and then go to bed. It’s just like that.” (Female, Malay #4)

#### Category IV: Patient-provider relationship

There were three themes identified in this category such as trusting healthcare providers, a desire to please healthcare providers, and fear of healthcare providers; which were reported by 70% of the patients in the study. Patients mentioned that they believe physicians are experts in the field, and they should therefore follow their advice in terms of treatment. One patient claimed that he would like to ‘help’ the physician by continuing to take his ADs because he appreciated the efforts of the physician who was trying to help him.

“I will follow the doctors’ advice. They are the professionals and I should follow what they have advised me. The doctors did tell me that this medicine is okay for me, so I just listen to what the doctors have said.” (Male, Chinese #3)

“The doctors have tried their best to help me, so I must help them back. So I just take it (ADs).” (Male, Indian #4)

#### Category V: Reminders

Reminders served as a cue that triggered patients to remember to take their medication. Using pillboxes, reminders from family members, and keeping medications in a visible place; were reported by patients as reminders for them to adhere to ADs.

“I have bought a pillbox. I take my pills according to the day written on the pillbox. I fill it by myself.” (Female, Malay #3)

“I will keep the medicine in places that [I] can easily see, so that I do not forget to take it. You see, it is always kept in my handbag wherever I go. I will not forget.” (Female, Malay #1)

#### Category VI: Social support networks

Social support networks refer to the sources of influence and encouragement that assisted patients in adhering to their ADs. These networks include family members, spouses, or co-workers. The majority of patients mentioned that spousal support motivated them to continue taking their medications.

“….my husband, he is very supportive. If there is no support from him I would not be sitting here talking to you. Because of his assistance and support, I have no problem taking my pill.” (Female, Chinese #4)

Having to assume responsibility for family members especially children, was most frequently cited by female patients as a motivating-factor to adhere to ADs.

“I am a single parent. I need to take care of my five children; I need to bring them up. I do not want my children to worry about my condition. I always remind myself I have five children to take care [of], [so] if I get sick, how am I going to take care of them? I know my children need me.” (Female, Malay #2)

## Discussion

### Barriers of adherence to antidepressants

The study found that almost all Malay and Chinese patients (80%) had erroneous beliefs or misconceptions towards MDD and its treatment, compared to only 50% of Indian patients. It was also noted that there were differences among the ethnicities with regard to reasons for non-adherence. Malay and Chinese patients tend to believe that they can stop taking their medications in the absence of depressive symptoms. They also believe that MDD would improve by having a positive mindset or complete rest instead of taking medications, which are perceived to be harmful. Our results are in keeping with the recent study conducted in Malaysia which found that 60% of patients with depression believed that they can take fewer tablets on days when they ‘feel better’ [42]. Another study in Malaysia also found that Chinese patients prefer alternative therapy, such as ‘sin sehs’ compared to Western medication; which could explain their more negative attitudes and beliefs toward ADs [43]. This is further augmented by the various cultural beliefs in Malaysia where the Malays believe that mental illness is an illness of the soul caused by weakness of the spirit, or as a social punishment to the sufferer [44]. In the Chinese culture, mental illness is believed to be caused by a lack of self-worthiness, which is measured by material achievement such as education and monetary gain that brings honour to the family. Indians on the other hand believe that evildoers could cast a spell on an individual to make them mentally ill [45]. All these unique cultural beliefs have led patients to seek guidance and help from traditional healers such as ‘bomohs’ or ‘sinsehs’ who are considered possessors of hidden knowledge to cure mental illnesses, instead of consulting mental healthcare providers [46].

The use of ADs is also hampered by patients’ negative beliefs and concerns regarding ADs. According to Horne’s theoretical model of medication adherence, patients’ decisions on adhering to medications are linked with their beliefs on the balance between the necessity, and concerns about the safety and efficacy of medicines being taken [47]. In the current study, most of the patients perceived that ADs are harmful, leading them to worry about damage to their kidneys as a result of long-term usage. These findings provide a preliminary insight that patients beliefs about the necessity for, and concerns about ADs, are the variables that account for their non-adherence [17, 20]. Hence, changing patients’ beliefs may help in improving their adherence.

The side effects of medications were highlighted by patients from all three ethnic groups as another significant concern that hampers their adherence to treatment. Similarly, patients suffering from other chronic diseases such as schizophrenia, diabetes, and hypertension; have also expressed their concerns regarding the safety of their medications, resulting in treatment discontinuation [48–51]. A cross-sectional survey conducted in the US reported that up to 80% of patients with schizophrenia experienced at least one side effect that was somewhat bothersome to them, causing them to discontinue their medications [48]. There is evidence that ADs such as Selective Serotonin Reuptake Inhibitors cause sexual dysfunction in both men and women, which may impair patients’ quality of life and self esteem, resulting in patients’ non-adherence to the prescribed ADs [23, 52, 53]. Hence, early identification of sexual side effects by patients, dosage titration to minimum effective doses, and choosing medications with safer side effect profiles, may improve patients’ adherence.

The study findings revealed that logistic barriers are the least reported (<10%) factors leading to non-adherence. In Malaysia, patients treated in public healthcare facilities are able to collect their medication refills at the nearest hospital or health clinic via SPUB (“Sistem Pendispensan Ubat Bersepadu”), which is one of the value-added services offered at all government hospitals and health clinic pharmacies [54]. SPUB is an integrated drug dispensing system implemented to enable patients who stay far away from their treatment facilities, to collect their medication refills from a public healthcare facility closer to their homes [55]. Thus, logistic barriers would not be an issue that hinder patients from adhering to their ADs. Besides that, stable patients can receive their follow-up treatment via the Community Psychiatric Unit, a community-oriented psychiatric service where care and treatment are delivered by a multidisciplinary team to patients’ homes; which is provided at most hospitals [46, 56]. Hence, the introduction of this service allows patients to receive treatment at home, subsequently solving the problem of frequent clinic visits.

### Facilitators of adherence to antidepressants

Illness-related factors such as having insight, is one of the key facilitators of medication adherence, as identified by patients in the study. Most of the patients were motivated to continue their ADs by a desire to achieve complete recovery and to prevent a relapse or recurrence. Similar findings were observed in studies involving patients with schizophrenia and bipolar disorder [57–59].

Besides having insight, it was found that 90% of patients continued their ADs as they perceived its health benefits in treating MDD. This included having positive beliefs about ADs, and the perceived effectiveness of ADs. According to the Common Sense Model (CSM) of illness self-regulation [60–62], it is proposed that illness beliefs or perceptions such as perceived health benefits of ADs, will lead to coping responses, which in turn influence health outcomes [63]. In this study, most of the patients continued their ADs because their depressive symptoms improved after initiating treatment. This in turn resulted in patients have positive beliefs about ADs, and agreeing to continue with treatment in the hope that it would help them return to baseline functioning.

It was noted that patients’ beliefs about medications are key barriers as well as facilitators of medication adherence. Perceived risk of having side effects, and perceived health benefits and positive attitudes towards therapy were common themes identified in this study. Although beliefs about medications are complex, it is nonetheless an important aspect to explore further and understand, in order to inform the design of interventions and policy solutions to enhance adherence in this group of patients. In addition, interventions may be more effective if it focuses on treatment-related attitudes and beliefs, particularly patients’ perceived benefits and necessity for treatment, rather than focusing on patients’ individual characteristics such as age or gender, which are characteristics that cannot be altered [64].

Patient-provider relationship was reported by 70% of patients as one of the facilitators of adherence, which was also found in several studies on adherence [27, 65–68]. Indeed, Asian patients tend to consider the physician as the authoritative source of knowledge and management for their illnesses, as ‘physicians were the only people who know best about their disease condition’ [69, 70]. Contrary to Asian patients, a study conducted in the US found that people in the West valued patient-physician partnerships via therapeutic alliances such as mutual trust among both parties, coordinated and continuous healthcare, and the patients’ perception of feeling respected and cared for [71, 72]. Unlike Asian countries, to be trustworthy, healthcare providers need to be able to provide optimal information, display a desire to promote the health of the patients, possess good interpersonal skills, show empathy with the patients’ condition, and give opportunities for patients to express their concerns [73, 74]. Thus, having a good patient-provider alliance via trustworthy healthcare providers would motivate patients to adhere to their treatment plan [75–78].

### Strengths and limitations

The main strength of this study is the use of face-to-face interviews to explore and gain an in-depth understanding on the underlying reasons for patients’ non-adherence /adherence to ADs. Furthermore, the sample population that comprised of a wide age range, equal gender representation, and ethnicity; allows for the generalizability of the study outcome to different populations. While this study was conducted in Malaysia, the findings are general with regard to barriers and facilitators, which can be applied to all populations. Besides that, populations in countries with similar religious backgrounds and collective cultures can also benefit from the findings of this study. There are relatively large numbers of Asian-born populations with similar cultural and religious backgrounds in countries such as the US (3.6%) [79], Australia (6.0%) [80] and Canada (9.8%) [81], and despite acculturation, many maintain or are still heavily influenced by the culture of their native countries [82, 83]. Hence, the study findings are generally applicable to these countries as well.

There are several limitations that need to be addressed in this study. Firstly, the study was conducted in an urban, government-run hospital in Malaysia where the participants have easy access to healthcare facilities, and healthcare charges are subsidized by the government. Therefore, the study findings may not be applicable to patients seeking treatment at healthcare facilities in rural areas, or in private settings where healthcare charges will be borne by patients. Secondly, patients were mostly from lower socioeconomic backgrounds with different educational levels, which could have affected patients’ comprehension of the questions being asked, subsequently affecting study findings. The exclusion of patients who could only communicate in Tamil may result in potential bias in the study outcomes.

### Implications for future research, policy and practice

This study reported on the barriers and facilitators of adherence in patients with MDD, which can be used as the basis for conducting large-scale multi-centre quantitative studies. Furthermore, the study findings could serve as an impetus for mental healthcare departments to set-up and provide funding for more adherence programs that aim to address the negative attitudes and beliefs in patients with MDD. This was proven in a study by Clifford et al [84] where a pharmacist-delivered intervention was implemented to address the concerns and beliefs of patients with chronic diseases regarding their medications, via a centralized telephone service to patients at home. This phone call intervention resulted in significantly more positive beliefs about medications (p = 0.007), and improved patients’ adherence to medications.

## Conclusions

This study revealed the barriers and facilitators of adherence to ADs in outpatients with MDD in a government-run healthcare setting in Malaysia. The study findings suggest that patient-specific barriers and medication side effects were the major challenges for adherence. Other barriers identified included healthcare provision and system, social-cultural, and logistics. Illness-related factors such as having insight and perceived health benefits on the need for treatment were the most frequently cited facilitators. Regular activities, patient-provider relationship, reminders, and social support networks; were also facilitators reported in the study. Taking into account patient-identified barriers and facilitators of adherence to ADs could help healthcare providers and policy makers to design targeted interventions that aim to tackle the key barriers, and promote measures to facilitate adherence in this group of patients.

## Supporting information

S1 File Interview questions for the study. (PDF)
