# Supplementary material for: Barriers and facilitators of adherence to antidepressants among outpatients with major depressive disorder: A qualitative study
Source: PLoS One. 2017 Jun 14;12(6):e0179290. doi: 10.1371/journal.pone.0179290 (PMC5470687; doi:10.1371/journal.pone.0179290)
Supplement: S1 File — (PDF) [file pone.0179290.s001.pdf]

SECTION A: DEMOGRAPHIC AND CLINICAL BACKGROUND DATA COLLECTION FORM

1. Age (years):

   _______ years old

2. Gender:
   - ○ Male
   - ○ Female

3. Ethnicity:
   - ○ Malay
   - ○ Chinese
   - ○ Indian

4. Religion:
   - ○ Islam
   - ○ Buddhist
   - ○ Hindu
   - ○ Christian
   - ○ Other ___________

5. Current marital status:
   - ○ Single
   - ○ Married
   - ○ Divorced
   - ○ Widowed
   - ○ Separated

6. Current household income/month (RM):
   - ○ < 1500
   - ○ 1501 – 2500
   - ○ 2501 – 3500
   - ○ 3501 - 4500
   - ○ >4500

7. Highest level of education you have completed:
   - ○ None
   - ○ Primary school
   - ○ Secondary school
   - ○ Diploma
   - ○ University or higher

8. Current employement:
   - ○ Unemployed
   - ○ Housewife
   - ○ Student
   - ○ Self-employed
   - ○ Retired
   - ○ Others __________________

9. When were you first diagnosed with depression?

   Year ___________

10. When were you first prescribed with antidepressant?

    Year ____________

11. What are the name and dose of antidepressants that you currently take?

(eg: T. Sertraline 50mg once a day) -*information obtained from patient's BHT*

___________________________________ ___________________________________

___________________________________ ___________________________________

___________________________________ ___________________________________

12. What other medical illnesses (co-morbid) do you have for which you take medication?

(eg: Hypertension, Diabetes, cancer, kidney failure, etc)- *Information obtained from patient's BHT*

___________________________________ ___________________________________

___________________________________ ___________________________________

___________________________________ ___________________________________

13. What other medication do you take apart from antidepressants?

Please state medication's name and dosage taken. (eg: T. Atenolol 100mg for hypertension)

-*information obtained from patient's BHT*

___________________________________ ___________________________________

___________________________________ ___________________________________

___________________________________ ___________________________________

___________________________________ ___________________________________

SECTION B: INTERVIEW GUIDE

**Background**	
1) Depression duration	How long have you had depression? *(……..months/years)*
2) Antidepressant duration	I understand that you are taking medication for your depression am I right? How long have you been taking it? *(…….mpnths/years)*
**Medications and Adherence**	
1) How it started	How do you take your antidepressant at home?
Probe	*Is anyone assisting you take your medication? (family/friends/care giver?)*
	How are they helping?
2) Influencing factors (barriers & facilitators)	What difficulties/problems do you face when taking your antidepressants as prescribed by your doctor?
	Do you have at any situations/factors that make it hard for you to take your antidepressant as prescribed by your doctor? *(if yes, can you describe the situation?)*
	What situations/factors make it easy for you to take your antidepressants as prescribed by your doctor?
3) Perception/view on antidepressants	How do you feel about your antidepressants?
	Do you plan to change the way you take your antidepressants? *(If yes, how and why?)*
4) Needs	What are your concerns about your antidepressants? What do you think could be done to help you take your medication? *(patient's point of view)*
Probe	*What can your family members do to help you?*
	What can you yourself do to help you take your antidepressants?
	How can healthcare providers (Dr./Ph./nurses) assist you to take your medication for depression?

	What are the ways that can help you take your antidepressants as prescribed by the doctor?
Probe	*What helps you remember to take your medication?*
**Beliefs and Experiences**	
1) Illness	Do you have any religious or cultural beliefs which might influence the way you feel about your depression? *(If yes, how & why? /If no, why no?)*
	How do your family and friends/co-worker feel about your depression?
2) Medication/treatment	Do you seek any spiritual or religious help for your depression? *(if yes, how & why? If no, why no?)*
	Have you ever stopped taking your antidepressants because of your religious or cultural beliefs? *(If yes, how & why?/If no, why no?)*
	Have you ever used/are you currently using herbal/traditional medicines for your depression? *(if yes, what & why?)*
**Wrap Up**	
	Do you have anything else to share regarding your depression or your antidepressants?
	Is there anything that you will like to ask me?
	How did you feel about this interview?

~END OF INTERVIEW~
